# Multiple adverse drug reactions during all-trans retinoic acid treatment for acute promyelocytic leukemia: differentiation syndrome, bradycardia, intestinal necrosis

**DOI:** 10.37349/etat.2020.00007

**Published:** 2020-04-28

**Authors:** Valeria Ferla, Mariarita Sciumé, Umberto Gianelli, Luca Baldini, Nicola Stefano Fracchiolla

**Affiliations:** 1 Hematology, Fondazione IRCCS Ca’ Granda-Ospedale Maggiore Policlinico, 20122 Milan, Italy; 2 Division of Pathology, Department of Pathophysiology and Transplantation, Fondazione IRCCS Ca’ Granda-Ospedale Maggiore Policlinico, 20122 Milan, Italy; 3 Department of Pathophysiology and Transplantation, Università degli Studi, Via Festa del Perdono 7, 20122 Milan, Italy; Istituto Nazionale Tumori “Fondazione Pascale” Via Mariano Semmola, Italy

**Keywords:** Acute promyelocytic leukemia, all-trans retinoic acid, adverse drug reaction

## Abstract

All-trans retinoic acid (ATRA) induces complete remission in a high proportion of acute promyelocytic leukemia (APL). Nevertheless it is be associated with adverse drug reactions that might be life-threatening including differentiation syndrome, myocarditis, myositis, Sweet’s syndrome and ulcers. We describe a case of APL who during induction therapy developed ATRA syndrome, cardiac arrhythmia and multiple episodes of intestinal necrosis that required surgery. In particular, we report here for the first intestinal necrosis attributable to ATRA treatment in the absence of histological evidence of promyelocytes infiltration or leukocytoclastic vasculitis.

## Introduction

Acute promyelocytic leukemia (APL) accounts for 10–15% of all cases of acute leukemia. APL is characterized by the reciprocal translocation between the long arm of chromosomes 15 and 17, causing the the fusion of the promyelocytic leukemia (PML)-promoter and the retinoic acid receptor (RAR)-α genes. The characteristic hemorrhagic coagulopathy is the principal responsible of the early death of these patients that may reach 29% of the cases. On the other hand, thromboses, both arterial and venous, are more common than in other acute leukemias, and occurr in 2% to 15% of the cases [1].

The PML-RAR-α transcript induces a cellular differentiation arrest. All-trans retinoic acid (ATRA), a derivative of vitamin A, binds to retinoic acid receptor, reactivating the transcription of crucial genes for differentiation [2], exerting its therapeutic activity. The combination of ATRA with anthracycline-based chemotherapy produces a complete remission rate (CR) close to 100% [3, 4]. Recent studies have demonstrated that low- or intermediate-risk APL can be cured in virtually all patients by a chemo-free combination of ATRA and arsenic trioxide (ATO) [5]. ATRA treatment is usually well tolerated, and the most frequent adverse events include fatigue, headache, fever, dermatitis, weakness, hypertriglyceridemia and gastrointestinal symptoms [2]. Major complications are rare and include differentiation syndrome, pseudotumor cerebri, myocarditis, myositis, Sweet’s syndrome and ulcers [6]. Here we report the clinical course of an APL patient characterized by the occurrence of multiple severe adverse drug reactions during induction APL therapy, including ATRA and chemotherapy.

## Case report

**Figure 1.**
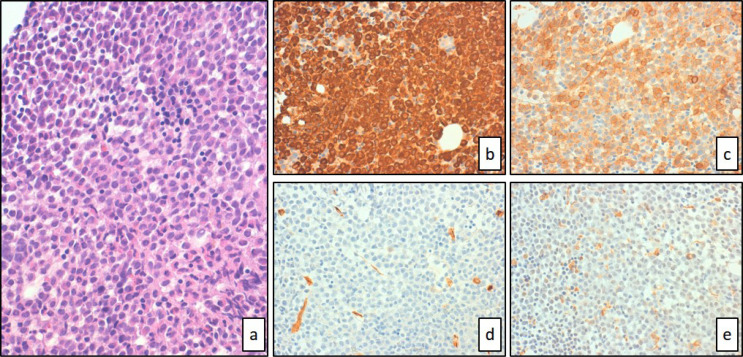
Bone marrow was subtotally occupied by diffuse sheets of hypergranular blasts with hematoxylin-eosin staining (a, 40× magnification). Immunohistochemical staining showed strong expression of MPO (b, 40× magnification) and CD117 (c, 40× magnification) but complete negativity for CD34 (d, 40× magnification) or CD68R (e, 40× magnification)

A 51-year-old woman was admitted at our center for metrorrhagia, asthenia and diffuse bone pain. At admission, laboratory analyses showed white blood cells 16.7 × 10^9^/L, hemoglobin 9 g/dL, platelets count 84 × 10^9^/L associated to a coagulation pattern indicative of overt disseminated intravascular coagulation (DIC) (prothrombin time as a ratio of 1.3 (normal, 0.88–1.21), partial thromboplastin time ratio 0.86 (normal, 0.86–1.20), D-dimer 42, 063 ng/mL (normal, 0–230), markedly reduced fibrinogen 89 mg/dL (normal, 165–350). Peripheral blood smear showed a massive presence of promyelocytes. Bone marrow morphological and histological analysis revealed a massive infiltration of typical promyelocytes (Figure 1). Cytogenetic analysis on peripheral blood demonstrated the presence of t(15;17) and molecular analysis showed the presence of PML-RAR-α fusion transcript, confirming the diagnosis of APL [7].

Bone marrow biopsy and aspirate revealed a hypercellular marrow dominated by sheets of promyelocytic-appearing blasts. Immunohistochemical studies showed blasts positivity for CD117+, CD13+,CD33+, CD38+CD2+CD56+, MPO+, CD11c+ (70% of blast population), CD15+ (50% of blast population), CD11b+/− (30% of blast population) and did not express CD3-CD7-CD14-CD19-CD34-CD79a-TdT-HLA-DR-.

The patient was classified as high risk APL.

The patient received supportive treatment, including fresh frozen plasma and platelet transfusions, to maintain platelet counts > 30 × 10^9^/L and fibrinogen levels > 150 mg/dL. Treatment with ATRA (45 mg/m^2^) divided in two daily doses PO in association with prednisone (0.5 mg/kg per day) was started.

At day 2 after ATRA treatment first administration, differentiation syndrome occurred, with rapid increase of white blood count (peak 49.9 × 10^9^/L), fever, respiratory distress, hypotension, peripheral edema associated to severe weight gain and diffuse bone pain requiring use of major opioids. ATRA was discontinued and high-dose dexamethasone plus diuretic therapy were promptly started with progressive clinical conditions improvement. Concurrently, the patient developed a severe bradycardia (33 bpm), effectively treated with i.v. atropine.

At day 3 the patient received idarubicin (12 mg/m^2^/d for 4 doses on days 2, 4, 6, and 8 of the induction phase) without acute complications. After the resolution of the differentiation syndrome, ATRA was resumed at 50% of the original dose for 7 days and thereafter at full dosage.

**Figure 2.**
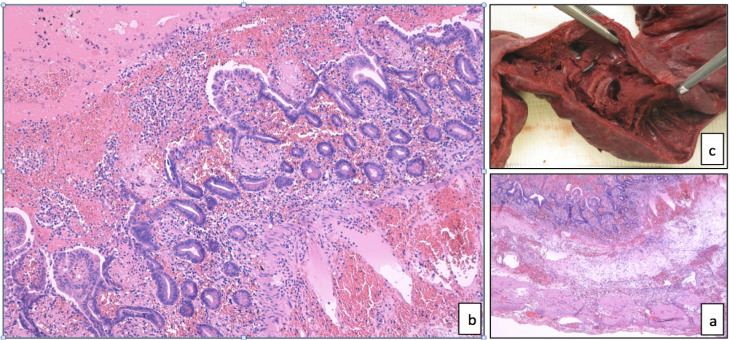
The infarcted small bowel (a, 4× magnification; b, 20× magnification) showed coagulative necrosis of the mucosa, edema and acute inflammation of the submucosa, associated to diffuse blood congestion and transmural. No evidence of promyelocytes infiltration was found in the gut or in the mesenteric fat, with Hematoxylin-eosin staining. The gross aspect of the bowel segment had already suggested the ischemic nature of the necrosis (c)

At day 13 a severe abdominal pain appeared. An abdominal computed tomography (CT) scan suggested an intestinal occlusion, at surgical inspection it was evidenced an ischemic segmentary ileitis and 30 cm of ileum was resected (Figure 2). In the following days, recurrent episodes of paroxysmal supraventricular tachycardia occurred, treated with adenosine and verapamil i.v., and a pre-excitation syndrome Wolff-Parkinson-White was diagnosed. Antiarrhythmic therapy with flecainide was started and subsequently associated with cardiac thermal ablation.

The recent major surgery and the recurrent episodes of paroxysmal supraventricular tachycardia suggested the possibility of the occurrence of acute pulmonary embolism: electrocardiography and echocardiography were uninformative, D-dimer was increased, and arterial blood gas test showed a mild hypoxemia and hypocapnia. A thoracic CT scan identified a pulmonary embolus in the dorsal segmental branch of the left upper lobe. Subcutaneous low molecular weight heparin was prescribed but it was immediately suspended because of melena occurrence. A CT scan performed six days later no longer detected venous perfusion defects. Inherited causes of thrombophilia were excluded, including deficiencies of antithrombin, protein S, and protein C, prothrombin gene mutations (FII G20210A), factor V Leiden and antiphospholipid antibodies.

Due to arrhythmic cardiac events, ATO therapy was considered contraindicated. Thirty days after the first dose, ATRA was restarted, but acute abdominal pain occurred after two doses. A second surgical ileum resection was performed following an abdomen CT scan, with histologic findings superimposable to previous resection (Figure 2).

In the post surgical period, the patient complained breath shortness and chest tightness and a chest X-ray showed significant pleural left effusion. One liter of pleural fluid was drained, with normal morphological, physico-chemical and cultural analyses (Table 1).

**Table 1.** Correlation between therapy, adverse events and hematological/biochemical parameters

**ATRA 45 mg/m^2^/d**	**Idarubicin 12 mg/m^2^/d**	**Day of induction therapy**	**Adverse events**	**WBC**	**N**	**PB**	**Hb**	**PLT**	**FD**	**D-D**	**PT**	**PTT**	**LDH**	**CRP**
Started		1		16.68	4.5	59	9	66	88	12429	1.27	0.97	2216	1.74
Interrupted		2	Differentiation syndrome	23.47	5.9	70	11.4	63	136	3633	1.07	0.77	1587	2.21
	1st Infusion	3	Bradycardia	49.90	5.99	79	10.2	25	117	34762	1.36	0.87	18440	8.38
		4		35.67	7.49	63	9	26	231	38773	1.24	0.79	16700	9
	2nd Infusion	5		19.86	6.16	52	9.5	39	189	38167	1.19	0.76	9592	12.27
Resumed at 50%		6		13.23	5.03	34	10.4	54	116	27330	1.15	0.73	5249	5.11
	3rd Infusion	7		5.98	2.09	28	10.6	48	67	17797	1.21	0.77	3456	1.7
		8		1.39	NE	NE	9.4	29	105	12442	1.19	0.74	2138	0.97
	4th Infusion	9		0.7	NE	NE	9.2	61	97	12203	1.2	0.72	1700	0.6
		10		0.4	NE	NE	8.5	45	126	9643	1.12	0.74	1330	0.42
		11		0.3	NE	NE	9.5	49	117	9021	1.14	0.77	1085	0.33
Resumed at 100%		12		0.15	NE	NE	9.6	69	124	6575	1.19	0.75	978	0.24
Interrupted		13	Ischemic segmentary ileitis	0.12	NE	NE	10.2	40	128	6273	1.14	0.72	893	0.53
		14		0.05	NE	NE	9.8	49	248	9265	1.36	0.87	800	4.4
		15	Paroxysmal supraventricular tachycardia	0.07	NE	NE	12.1	50	382	8559	1.38	0.92	710	29.98
		16		0.05	NE	NE	11.1	24	490	5191	1.45	0.93	641	43.48
		17	Pulmonary emboly	0.06	NE	NE	12.3	33	786	4902	1.32	1.04	532	40.71
		18		0.09	NE	NE	11	41	799	3345	1.12	0.91	498	35.45
		19		0.1	NE	NE	11	38	430	6565	1.05	0.75	494	13.78
		20		0.21	NE	NE	11	18	279	4496	1.23	0.85	623	6.65
Resumed at 100%		30	Ischemic segmentary ileitis	3.42	3.1	0	8.7	261	580	1535	1.16	0.92	550	5.1
Interrupted		31	Differentiation syndrome	3.78	3.36	0	9.7	291	449	1230	1.12	0.96	532	4.96

WBC: white blood cells, 10^9^/L; N: neutrophils, 10^9^/L; PB: peripheral blasts, %; Hb: hemoglobin, g/dL; PLT: platelets, 10^9^/L; FD: fibrinogen, mg/dL; D-D: D-dimer, ng/mL; PT: prothrombin time; PTT: partial thromboplastin time; LDH: lactic dehydrogenase, U/L; CRP: C-reactive protein, g/dL; NE: not evaluable

Forty days after the first administration of ATRA, bone marrow evaluation showed morphologic and molecular CR. Three months after diagnosis, the patient was in CR and the first cycle of consolidation was administered (cytarabine 1 g/m^2^/d and idarubicin 5 mg/m^2^/d on days 1-2-3-4, ATRA 45 mg/m^2^/d in two equally divided doses). The patient refused consent to perform diagnostic lumbar puncture. A brain CT scan showed normal findings.

The consolidation cycle was interrupted after the first day because of severe abdominal pain and fever. Abdomen CT scan showed free intraperitoneal air suggestive for intestinal perforation. Antibiotic therapy (meropenem and piperacillin/tazobactam) was started, with significant clinical improvement and inflammatory markers normalization.

Following the life-threatening adverse events during the therapy and the persistence of molecular CR a watch and wait approach was adopted. Eight months later, the patient reported the appearance of persistent headache and loss of strength in the right leg. The cerebrospinal fluid morphological examination showed leukemic infiltration. Intrathecal therapy with cytarabine, methotrexate and dexamethasone and ATO intravenously for 4 courses were performed, followed by a whole-brain and spinal irradiation for a total dose of 24 Gy. ATRA was not reintroduced in therapy. Seven years after diagnosis, the patient is still alive and well, with persistent APL CR.

## Discussion

ATRA syndrome occurs in 25% of APL patients treated with ATRA monotherapy, and in 10–15% of patients treated with ATRA and chemotherapy combinations. It usually develops within the first 3 weeks of treatment, with a median of 7–12 days from the first dose. The pathogenesis is correlated to leukocyte infiltration and cytokine release which determine endothelial damage and extravasation of APL cells following ATRA induced blast maturation [8–13].

Our patient, affected by high-risk APL, rapidly developed ATRA syndrome after three ATRA doses, with the classic clinical picture characterized by a rapid increase in white blood count, dyspnea, body weight gain and diffuse bone pain, with a rapid response to dexamethasone administration.

Bradyarrhythmia is a rare adverse event during ATRA treatment, previously described only in two cases. McGregor et al. [14], described bradycardia in a patient receiving ATRA and idarubicin, following ATRA syndrome occurrence. Maruhash et al. [15], described a patient who developed bradycardia after three days of ATRA therapy, which did not improve with medical therapy and worsened following ATRA re-challenge. This suggests that ATRA may contribute to sinus node arrhythmia, causing, or exacerbating pre-existing node dysfunctions, with mechanism poorly anderstood.

In our patient, bradycardia was followed by supraventricular tachycardia in the context of a previously undiagnosed Wolff-Parkinson-White syndrome. Recurrent intestinal necrosis represents the most severe adverse event occurred in our case, requiring two limited bowel resections. Anedoctical similar cases are described in literature, with mechanisms poorly understood. Farah et al. [16], described a child who developed multiple splenic, renal, and intestinal infarctions during the course of induction chemotherapy in association with ATRA, possibly related to acquired protein C deficiency. Acquired protein C deficiency is correlated with different clinical conditions, as severe infection, liver disease, DIC and acute respiratory distress syndrome, the latter two conditions occurring in our patient. Yamada et al. [17], reported a case of ileum necrosis attributed to ATRA-induced leukocytoclastic vasculitis. Bhargava et al. [18], described a patient who presented an early differentiation syndrome after a single ATRA dose and developed ileal perforation associated with massive promyelocytes infiltration of the bowel. A possible mechanism proposed is the induction of adhesion molecules expression mediated by ATRA on APL blasts during differentiation, enhancing multiple tissues, such as intestinal wall [18].

In our patient, both ileum necroses presented a close temporal correlation with the ATRA administration (during the first two weeks of therapy and at re-challange), strongly supporting a causal relationship. Furthermore, we were able to demonstrate histologically an infiltration of leukemic promyelocytes of intestinal tissue, while a significant infiltrate of inflammatory cells was evident, that might have represented the pathogenetic effectors of ischemia and subsequent perforation. Finally, according to Narajo algorithm, the occurrence of ileum necrosis in our patient was “definitely” correlated to an adverse drug reaction (ADR) of ATRA therapy (score ≥ 9 points) [19], and was “unpreventable” according to modified Schumock and Thornton criteria [20].

In conclusion, we have described the rare occurrence of multiple and severe ADRs in an APL patient, successfully treated with prompt administration of specific medical therapy and emergency surgery, in a multidisciplinary setting. Their occurrence in rapid sequence after the administration of ATRA may suggest that the differentiation syndrome-related cytokine storm, induced a hyper-acute inflammatory status that contributed to the pathogenesis of brady- and tachy-arrythmias, thromboembolic events and intestinal ischemia with necrosis.

## Abbreviations

APL: acute promyelocytic leukemia
ADR: adverse drug reaction
ATO: arsenic trioxide
ATRA: all-trans retinoic acid CR: complete remission rate CRP: C-reactive protein
CT: computed tomography
D-D: D-dimer
DIC: disseminated intravascular coagulation
Hb: hemoglobin
LDH: lactic dehydrogenase
PB: peripheral blasts
PML: promyelocytic leukemia
PT: prothrombin time
PTT: partial thromboplastin time
WBC: white blood cells

## Declarations

### Copyright

© The Author(s) 2020.

## References

[1] Breccia M Lo Coco F. Thrombo-hemorrhagic deaths in acute promyelocytic leukemia. Thromb Res. 2014;133 Suppl 2:S112–6. 10.1016/S0049-3848(14)50019-9 24862130

[2] Ipec Y Hulya D Melih A. Disseminated exfoliativedermatitis associated with all-transretinoic acid in the treatment of acute promyelocytic leukemia. Case ReportsMed. 2012;2012:236174. 10.1155/2012/236174 PMC3405663 22848226

[3] Fenaux P Chastang C Chevret S Sanz M Dombret H Archimbaud E A randomized comparison of all transretinoic acid (ATRA) followed by chemotherapy and ATRA plus chemotherapy and the role of maintenance therapy in newly diagnosed acute promyelocytic leukemia. Blood. 1999;94:1192–200. 10438706

[4] Tallman MS Andersen JW Schiffer CA Appelbaum FR Feusner JH Ogden A All-trans-retinoic acid in acute promyelocytic leukemia. N Engl J Med. 1997;337:1021–8. 10.1056/NEJM199710093371501 9321529

[5] LoCoco F Avvisati G Vignetti M Thiede C Orlando SM Iacobelli S Retinoic acid and arsenic trioxide for acute promyelocytic leukemia. N Engl J Med. 2013;369:111–21. 10.1056/NEJMoa1300874 23841729

[6] Fenaux P De Boutton S. Retinoic acid syndrome.Recognition, prevention and management. Drug Saf. 1998;18:273–9. 10.2165/00002018-199818040-00003 9565738

[7] Sanz MA Lo Coco F Martín G Avvisati G Rayón C Barbui T Definition of relapse risk and role of nonanthracycline drugs for consolidation in patients with acute promyelocytic leukemia: a joint study of the PETHEMA and GIMEMA cooperative groups. Blood. 2000;96:1247–53. 10942364

[8] Frankel SR Eardley A Lauwers G Weiss M Warrell RP Jr. The “retinoic acid syndrome” in acute promyelocytic leukemia. Ann Intern Med. 1992;117:292–6. 10.7326/0003-4819-117-4-292 1637024

[9] Holmes D Vishnu P Dorer RK Aboulafia DM. All-trans retinoic acid-induced pseudotumor cerebri during induction therapy for acute promyelocytic leukemia: a case report and literature review. Case Rep Oncol Med. 2012;2012:313057. 10.1155/2012/313057 22701192 PMC3371673

[10] Choi S Kim HS Jung CS Jung SW Lee YJ Rheu JK Reversible symptomatic myocarditis induced by all-trans retinoic acid administration during induction treatment of acute promyelocytic leukemia: rare cardiac manifestation as a retinoic acid syndrome. J Cardiovasc Ultrasound. 2011;19:95–8. 10.4250/jcu.2011.19.2.95 21860725 PMC3150704

[11] Citak FE Ezer U Akkaya E Ozbulbul N Bahce M Kurekci AE. All-trans-retinoic acid-induced myositis in a child with acute promyelocytic leukemia. Haematologica. 2006;91 Suppl 8:ECR35. 16923519

[12] Park CJ Bae YD Choi JY Heo PS Lee KS Park YS Sweet’s syndrome during the treatment of acute promyelocytic leukemia with all-trans retinoic acid. Korean J Intern Med. 2001;16:218–21. 10.3904/kjim.2001.16.3.218 11769583 PMC4531725

[13] Shimizu D Nomura K Matsuyama R Matsumoto Y Ueda K Masuda K Scrotal ulcers arising during treatment with all-trans retinoic acid for acute promyelocytic leukemia. Intern Med. 2005;44:480–3. 10.2169/internalmedicine.44.480 15942099

[14] McGregor A Hurst E Lord S Jones G. Bradycardia following retinoic acid differentiation syndrome in a patient with acute promyelocytic leukaemia. BMJ Case Rep. 2012;2012:bcr0220125848. 10.1136/bcr.02.2012.5848 PMC3417020 22778455

[15] Maruhashi K Wada H Taniguchi M Koizumi S. Sinus bradyarrhythmia during administration of all-trans retinoic acid in a patient with acute promyelocytic leukemia. Rinsho Ketsueki. 1996;37:443–7. Japanese. 8691592

[16] Farah RA Jalkh KS Farhat HZ Sayad PE Kadri AM. Acquired protein C deficiency in a child with acute myelogenous leukemia, splenic, renal, and intestinal infarction. Blood Coagul and Fibrinolysis. 2011;22:140–3. 10.1097/MBC.0b013e32834248e6 21178585

[17] Yamada K Sugimoto K Matsumoto T Narumi K Oshimi K. All-trans retinoic acid-induced vasculitis and hemonecrosis of the ileum in a patient with acute promyelocytic leukemia. Leukemia. 1999;13:647–8. 10.1038/sj.leu.2401379 10214877

[18] Bhargava R Dolai TK Singhal D Kumar R Pathak P. Retinoic acid syndrome after first dose of ATRA and ileal perforation secondary to promyelocytes infiltration. Leuk Res. 2008;32:997–8. 10.1016/j.leukres.2007.09.021 18022229

[19] Naranjo CA Busto U Sellers EM Sandor P Ruiz I Roberts EA A method for estimating the probability of adverse drug reactions. Clin Pharmacol Ther. 1981;30:239–45. 10.1038/clpt.1981.154 7249508

[20] Schumock GT Thornton JP. Focusing on the preventability of adverse drug reactions. Hosp Pharm. 1992;27:538. 10118597

